# The management of functional dyspepsia in clinical practice: what lessons can be learnt from recent literature?

**DOI:** 10.12688/f1000research.12089.1

**Published:** 2017-09-28

**Authors:** Maura Corsetti, Mark Fox

**Affiliations:** 1 National Institute for Health Research, Nottingham Digestive Diseases Biomedical Research Centre, Nottingham University Hospitals NHS Trust , University of Nottingham, Nottingham, UK; 2 Abdominal Centre: Gastroenterology, St Claraspital, Basel, Switzerland; 3 Clinic of Gastroenterology & Hepatology, University Hospital Zürich, Zürich, Switzerland

**Keywords:** gastrointestinal disorder, functional dyspepsia, gastroenterology

## Abstract

Functional dyspepsia is a prevalent functional gastrointestinal disorder that can significantly erode the quality of life of sufferers and places a major cost burden on healthcare services. In this article, we review the recent literature, selecting the information we consider relevant since it has changed our clinical management of patients with functional dyspepsia.

## Introduction

About 20% of the population has chronic symptoms that can be attributed to disorders of gastroduodenal motility and function, which the recently published Rome IV criteria have classified into four categories: functional dyspepsia (FD), belching disorders, chronic nausea and vomiting disorders, and rumination syndrome ^1^. This diagnostic classification is largely unchanged from previous iterations with only minor changes made to the definition ( Table 1).

**Table 1.** Rome IV criteria for functional dyspepsia.

Diagnostic Rome IV criteria for functional dyspepsia (changes from Rome III criteria appear in bold) 1. One or more of the following: a. bothersome postprandial fullness; b. bothersome early satiation; c. bothersome epigastric pain; d. bothersome epigastric burning AND 2. No evidence of structural disease (including at upper endoscopy) that is likely to explain the symptoms *Criteria fulfilled for the last 3 months with symptom onset at least 6 months before diagnosis
Diagnostic criteria for postprandial distress syndrome Must include one or both of the following **at least 3 days per week**: 1. **Bothersome** postprandial fullness ( **that is, severe enough to impact on usual activities**) 2. **Bothersome** early satiation ( **that is, severe enough to prevent finishing a regular-sized meal**) *Supportive criteria* 1. Postprandial epigastric pain or burning, epigastric bloating, excessive belching, and nausea can also be present; 2. **vomiting** **warrants consideration of another disorder**; 3. **heartburn is not a dyspeptic symptom but may often co-exist**; 4. **symptoms** **that are relieved by evacuation of faeces or gas should generally not be considered part of dyspepsia**; 5. **other individual** **digestive symptoms or groups of symptoms (such as from gastro-oesophageal reflux disease and irritable bowel syndrome)** **may co-exist with postprandial distress syndrome.**
Diagnostic criteria for epigastric pain syndrome Must include at least one of the following symptoms at least 1 day a week: 1. **Bothersome** epigastric pain ( **that is, severe enough to impact on usual activities**) 2. **Bothersome** epigastric burning ( **that is, severe enough to impact on usual activities**) *Supportive criteria* 1. Pain may be induced by ingestion of a meal, may be relieved by ingestion of a meal, or may occur while fasting; 2. postprandial epigastric bloating, belching, and nausea can also be present; 3. **persistent vomiting likely suggests another disorder**; 4. **heartburn is not a dyspeptic symptom but may often co-exist**; 5. the pain does not fulfil biliary pain criteria; 6. symptoms that are relieved by evacuation of faeces or gas generally should not be considered part of dyspepsia; 7. **other digestive symptoms** **(such as from gastro-oesophageal reflux disease and irritable bowel syndrome) may co-exist with epigastric pain syndrome.**

## Definition

FD is a functional gastrointestinal (GI) disorder characterized by one or more of the following symptoms: postprandial fullness, early satiation, epigastric pain, and epigastric burning ^1^. Nausea and vomiting are no longer considered cardinal FD symptoms and have been moved into separate categories of functional nausea and vomiting disorders ^2^. Based on factor analyses of digestive symptoms in the general population and FD patients, the Rome Committee further divided patients with FD into two subgroups: postprandial distress syndrome (PDS), which is characterized by meal-induced symptoms, and epigastric pain syndrome (EPS), which refers to epigastric pain or epigastric burning that does not occur exclusively postprandially and can even be improved by meal ingestion ^2^. In clinical practice, PDS is more prevalent than EPS ^1^; however, the overlap between PDS and EPS patients has been reported to be as high as 50% ^1, 2^. Therefore, the clinical utility of this subdivision has been questioned ^1^, even though it seems reasonable to distinguish between patients with symptoms induced by food intake (that is, PDS, previously called “dysmotility-like”) and those in whom symptoms are largely independent of GI function (that is, EPS, previously called “ulcer-like”). Moreover, a recent study has demonstrated that the overlap can be reduced by a more rigorous linking of postprandially occurring symptoms to PDS, regardless of their qualitative nature ^3, 4^. This means that epigastric pain or burning, if postprandial, can also be present in PDS ^3, 4^. One change made by Rome IV is to limit the diagnosis of FD to patients with “bothersome symptoms… severe enough to impact on usual activities” occurring with a minimal frequency of 3 days per week ^1^. This is necessary to distinguish those with clinically relevant disease from healthy subjects with occasional, relatively mild symptoms that can be considered part of normal daily life. FD patients who meet these diagnostic criteria have reduced quality of life and incur significant direct costs through medical expenses and indirectly through loss of productivity ^1^.

## Pathophysiology

The causes of FD are not completely understood; however, several mechanisms appear to be involved. These include altered gastric emptying, impaired gastric accommodation, gastric and duodenal hypersensitivity, and previous GI infections ^1^. Additionally, the presence of psychiatric disease and psychosocial stress factors has a role in FD aetiology and severity ^1^. Delayed gastric emptying has been reported in up to 35% of patients with FD, whereas rapid gastric emptying, even if less studied, is probably present in a lower percentage of patients ^1^. Impaired gastric relaxation in response to food (that is, impaired accommodation) has also been found in about one third of patients with FD ^1^. Hypersensitivity to mechanical stimulation (for example, distention) of the stomach or the small bowel (or both) is frequent, and affected patients may also show hypersensitivity to chemical stimuli such as acid and lipids ^1^. Acute infection is known to trigger so-called “post-infectious FD” in at least 10–20% of individuals ^1^. The diagnosis of “ *Helicobacter pylori*-associated dyspepsia” can be made if successful eradication leads to long-term resolution of symptoms ^1^. A relationship between the gut and psyche has been described in this condition because patients with psychological disease (that is, anxiety or depression) are at increased risk of developing FD, and vice versa ^1^. Many of these factors can impact on mucosal integrity and duodenal permeability, effects that have been related to the activation of the innate mucosal immune system. Experimental evidence provides some insight into this complex mechanism in humans. Psychological stress mediated by corticotrophin-releasing hormone has been shown to alter intestinal permeability through a mast cell–dependent mechanism ^5^. Recent research has also shown differences in the duodenal microbiome between patients with FD and controls, although it remains uncertain whether this represents “cause or effect”.

Studies have also evaluated whether the two FD subgroups—PDS and EPS—differ with regard to pathophysiological mechanisms. The results suggest that PDS is associated with gastric hypersensitivity, delayed gastric emptying, anxiety, and the presence of increased intra-epithelial eosinophils in the duodenum and other evidence of an activated mucosal immune system ^6– 9^. Additionally, PDS presents frequently “as an overlap syndrome” with other functional GI disorders (for example, irritable bowel syndrome, or IBS) ^10^. In contrast, patients with EPS have little evidence of abnormal GI motility and function. EPS may be associated with the presence of other centrally mediated pain syndromes (for example, fibromyalgia) ^11^.

## Diagnosis

The diagnosis of FD is based on clinical assessment and exclusion of organic disease. Thus, before the diagnosis of FD is made, laboratory tests and upper GI endoscopy with biopsies are normally performed to exclude infection (in particular, *H. pylori*), peptic ulceration, celiac disease, and neoplasia ^1^. Physiological investigations are normally not required at this stage ^1^.

### Differential diagnosis

Gastro-oesophageal reflux disease (GERD) is a potential cause of “dyspeptic symptoms” that is highly prevalent in the community. GERD is present when the reflux of stomach contents causes symptoms or mucosal disease (or both) and is characterized by the presence of heartburn and regurgitation. Formally, if heartburn is present, then this excludes the diagnosis of FD in the Rome IV classification ^12^. However, studies show that reflux and dyspeptic symptoms co-exist in up to 40% of cases, which is much more often than expected by chance ^13– 15^. This overlap between GERD and FD is not surprising given the immediate proximity and shared innervation of the oesophagus and proximal stomach.

Rome IV divides patients with GERD into those with erosive reflux disease (ERD), non-ERD (NERD, pathological acid exposure), reflux hypersensitivity (RH, normal acid exposure but association between reflux and symptoms), and functional heartburn (FH, no association between reflux and symptoms) ^12^. FD overlaps with ERD, NERD, RH, and FH with increasing frequency. Patients with FH have more frequent PDS symptoms, whereas patients with NERD have more EPS symptoms (typically, epigastric burning) ^15, 16^. Conversely, pathological acid exposure is present in about one third of patients with a clinical diagnosis of FD and in at least half of the subgroup with epigastric burning, which also more frequently responds to proton pump inhibitor (PPI) therapy ^17^. Not only oesophageal but also duodenal acid exposure has been linked to EPS. Acid exposure of the duodenum is also associated with duodenal infiltration with inflammatory cells in patients with FD ^18^. Mucosal acid exposure is a possible mechanism in duodenal permeability, predisposing to duodenal inflammation and altered sensitivity ^19^. These observations may well explain why a proportion of FD patients without GERD respond to acid suppression. It is clear that several pathologies can cause symptoms, and conversely it is very likely that the overlap between the two functional conditions is the result of the shared pathophysiological mechanisms (for example, hypersensitivity) playing a role in two different regions of the GI tract. If the diagnosis remains uncertain after a trial of acid suppression, then manometry and ambulatory pH-impedance studies provide an objective assessment of oesophageal acid exposure and the association between reflux and patient symptoms ^12^. Additionally, these tests can identify dysmotility (for example, oesophageal spasm) that can present with similar symptoms and behavioural conditions (for example, rumination syndrome) that are frequently triggered by dyspeptic symptoms ^12^.

FD can also overlap with chronic nausea and vomiting disorders. According to Rome IV criteria, these disorders include chronic nausea and vomiting syndrome (CNVS), cyclic vomiting syndrome (CVS), and cannabinoid hyperemesis syndrome (CHS) ^1^. CNVS is characterized by the presence of bothersome (that is, severe enough to impact on usual activities) nausea, occurring at least 1 day per week and/or one or more vomiting episodes per week in the absence of an organic explanation of symptoms or of self-induced vomiting, eating disorders, regurgitation, or rumination. CVS is characterized by stereotypical episodes of vomiting regarding onset (acute) and duration (less than 1 week) with at least three discrete episodes in the prior year and two episodes in the past 6 months, occurring at least 1 week apart in the absence of vomiting between episodes. CHS is characterized by episodes resembling CVS in terms of onset, duration, and frequency; presents after prolonged excessive cannabis use; and is relieved by sustained cessation of cannabis use. These disorders have been, in general, poorly studied, and it is possible that they overlap with other functional disorders. Given the current knowledge about these disorders, it is necessary to identify patients with nausea or vomiting (or both) as prevalent symptoms, as these patients are more difficult to manage in clinical practice and probably should be referred to specialized centres.

IBS is a frequent functional disorder with a prevalence of about 10% in the general population. Rome IV defines the condition in terms of the presence of chronic recurrence of pain in association with defaecation and/or an altered bowel habit in terms of either stool frequency or stool consistency ^20^. An important overlap is present between FD and IBS with both occurring in 30% and 60% of patients, respectively ^10^. Overlap may be more common in patients with severe symptoms than in patients with mild symptoms and in PDS than in EPS, especially in patients with postprandial fullness ^10, 21, 22^. Moreover, longitudinal studies demonstrate that the risk of a patient with FD developing IBS is increased by up to eightfold compared with the general population ^23^. These findings have led many to question whether these conditions are truly distinct or share common pathogenic mechanisms. Indeed, the pathophysiological mechanisms shared by PDS and IBS are very similar and include anxiety, altered motility, and visceral hypersensitivity. Low-grade inflammation with activation of the innate mucosal immune system and increased mucosal permeability has also been documented in both conditions ^19^.

## Management

The management of patients with FD should start with reassurance and education about the possible pathophysiological and risk factors associated with FD appropriate for the PDS or EPS subgroup or both ^1^. Lifestyle and dietary recommendations may be helpful ^1, 24^. Avoidance of non-steroidal anti-inflammatory drugs, coffee, high-fat foods, alcohol, and smoking is commonly recommended on the basis of physiological studies and case reports; however, the real value of these recommendations is unclear ^1, 24^.

The next step recommended by the Rome committee is to exclude iatrogenic causes of dyspeptic symptoms and to recognize and treat overlapping disease. Identification of *H. pylori* infection is appropriate, as prospective trials indicate that eradication therapy is curative in approximately 1 in 10 infected patients ^1^. If the patient is not infected, then an empirical trial of acid suppression is justified to suppress symptoms related to an atypical presentation with GERD ^1^. Individuals with overlap IBS may respond to spasmolytics and stool regulation.

Pharmacological treatments for FD that are more effective than placebo in randomized controlled trials and are available in the market are limited ^1, 24^. These include acid suppression (PPIs), H2 receptor antagonists (H2RAs), prokinetics, herbal preparations, and antidepressants ^1, 24^. Dietary interventions and medications that modulate digestive function may be more likely to be effective in PDS patients in whom abnormal gastric function is present ^1^. Conversely, pain modulators and, if appropriate, antidepressants may be most appropriate in EPS ^1^. This hypothesis is (indirectly) supported by the observation that the presence of normal gastric emptying on scintigraphy in patients with FD is associated with a good response to low-dose antidepressant medications that target visceral hypersensitivity (see below).

### Acid and reflux suppression

A just-published Cochrane systematic review has concluded that PPIs are effective for the treatment of FD, independent of the dose and duration of treatment compared with placebo. PPIs may be slightly more effective than H2RAs for the treatment of FD, even if the evidence is scarce ^25^. A recent randomized, placebo-controlled trial with an alginate-antacid preparation (Gaviscon) that controls both acid and non-acid reflux has also shown a significant benefit not only in typical reflux but also in dyspeptic (epigastric pain) symptoms ^26, 27^. It is uncertain what proportion of patients who respond to acid and reflux suppression have an atypical presentation of GERD.

### Prokinetics

Historical studies with cisapride, a mixed 5-HT4 agonist and 5-HT3 antagonist with procholinergic effects, indicate that selected prokinetics can be more effective than placebo in treating FD ^24^. Unfortunately, this medication is now restricted in most countries because of increased risk of tachyarrhythmia in patients with heart disease ^24^. Only limited data are present for the dopamine-2 antagonists domperidone and metoclopramide although they are prescribed extensively ^24^. However, owing to cardiac and neurological side effects, the use of these medications for long-term treatment is not recommended. One phase IIb randomized, placebo-controlled study reported that itopride, a dopamine D2 antagonist and acetylcholinesterase inhibitor, is effective in FD, in particular for the management of pain and fullness ^24^. However, two subsequent phase III trials were negative ^24^. Whether this was related to the selection of different patient populations in the original Japanese and the subsequent US trials remains uncertain. More recent data have demonstrated that acotiamide at a dose of 100 mg three times daily was efficacious and safe in the treatment of PDS ^28– 30^. The drug has been commercially available in Japan since 2013, and trials in Europe and the USA are in progress ^28^. Interestingly, a higher percentage of patients with PDS have been reported to respond to the treatments with acotiamide. It may be that this is related to effects on gastric motility and gastric emptying documented in animal models ^28^. Data have also recently appeared about the possible effect of prucalopride, a 5-HT4 agonist licensed in Europe and Canada for the treatment of refractory constipation, in treating FD. This drug increases oesophageal and gastric motility in healthy subjects ^31^, and recent data, still in abstract form, have also reported a benefit in treating symptoms of patients with FD and gastroparesis ^32^. Iberogast (STW5), a nine-herb combination, has been shown in studies to relax the gastric fundus, promote gastric emptying, and reduce visceral sensitivity through multiple putative mechanisms ^1^. Some clinical data also support its use, and it is a popular over-the-counter remedy for FD in several European countries. However, a recent report of severe hepatotoxicity leading to liver transplantation potentially associated with the use of Iberogast suggests some caution in prescribing this medication ^33^. Finally, rikkunshito, another herbal medicine, which is thought to accelerate gastric emptying, has been shown to improve symptoms of epigastric pain and postprandial fullness in patients with FD in a randomized clinical trial ^34^.

### Centrally acting drugs

A substantial body of work supports the use of low-dose antidepressants in the management of FD and other functional GI disorders and chronic pain syndromes ^35^. Talley *et al*. ^36^ recently conducted an important randomized controlled trial that compared the effects of two classes of antidepressant in FD: (i) the tricyclic amitriptyline (50 mg) and (ii) the selective serotonin reuptake inhibitor (SSRI) escitalopram (10 mg). A large number (n = 292) of patients with Rome II dysmotility-like (similar to PDS) or ulcer-like (similar to EPS) FD were studied. Solid gastric emptying was documented by gastric scintigraphy, and the maximally tolerated ingestion of liquid nutrient was documented to estimate gastric accommodation and sensation ^36^. Significant treatment effects were observed over 12 weeks. Amitriptyline, but not escitalopram, appeared to benefit patients with FD, particularly those with ulcer-like FD (EPS-like) ^36^. Interestingly, delayed gastric emptying was associated with a poor response to treatment, a finding that supports the hypothesis that clinical presentation and physiological studies can help direct therapy ^36^. These findings are consistent with the Nortriptyline for Idiopathic Gastroparesis (NORIG) study, which randomly assigned 130 patients with idiopathic gastroparesis to nortriptyline (a tricyclic antidepressant) or placebo for 15 weeks (91% of patients met diagnostic criteria for PDS) ^37^. In this study, active treatment was not superior to placebo in treating patients; however, the majority (86%) had gastroparesis on scintigraphy ^37^. Importantly, the trial by Talley *et al*. found that, although adverse events were commonly reported, there was no difference in side effects among the placebo, amitriptyline, and escitalopram, except in neurological symptoms with the SSRI ^36^. These findings support the use of amitriptyline in FD patients without delayed gastric emptying. A course of therapy without documenting gastric emptying can be justified, as these medications are generally well tolerated at low doses.

A just-published meta-analysis concluded that certain neuroleptics, like levosulpiride, have prokinetic actions and are more effective than placebo in patients with FD in secondary or tertiary care ^35^. However, it remains uncertain whether other psychotropic drugs, including 5-HT1A receptor agonists, tetracyclic antidepressants, or serotonin-norepinephrine reuptake inhibitors (SNRIs), are effective treatments in FD ^35^. In the case of 5-HT1A agonists, although there have been three trials, each used a different drug (sumatriptan, buspirone, and tandospirone), and the results were conflicting ^35^. These medications are of particular interest because, in addition to their anxiolytic and antidepressant effects, both relax the gastric fundus and reduce gastric sensitivity (key pathological mechanisms in PDS). Trials reported reduced symptoms of postprandial fullness, early satiation, and upper abdominal bloating in randomized, placebo-controlled trials ^35^. Unfortunately, although these medications are helpful in some individuals, sedative side effects limit their use in others. In the case of tetracyclic antidepressants and SNRIs, there has been only one trial of each of these drug classes (mirtazapine and venlafaxine) ^35^.

### Psychological and other interventions

Controlled trials suggest clinical benefit of psychological interventions from several, small randomized controlled studies ^38^; however, the quality of evidence is still suboptimal. A recent systematic review concluded that acupuncture therapy achieves a statistically significant effect for FD in comparison with sham acupuncture and is superior to medication (prokinetic agents) in improving the symptoms and quality of life of patients with FD ^39^. Nonetheless, despite stringent methodological analyses, there is still need for additional randomized controlled studies of higher quality ^39^.

## Is this information from the literature of any relevance for an evidence-based approach in our clinical practice?

**Figure 1.**
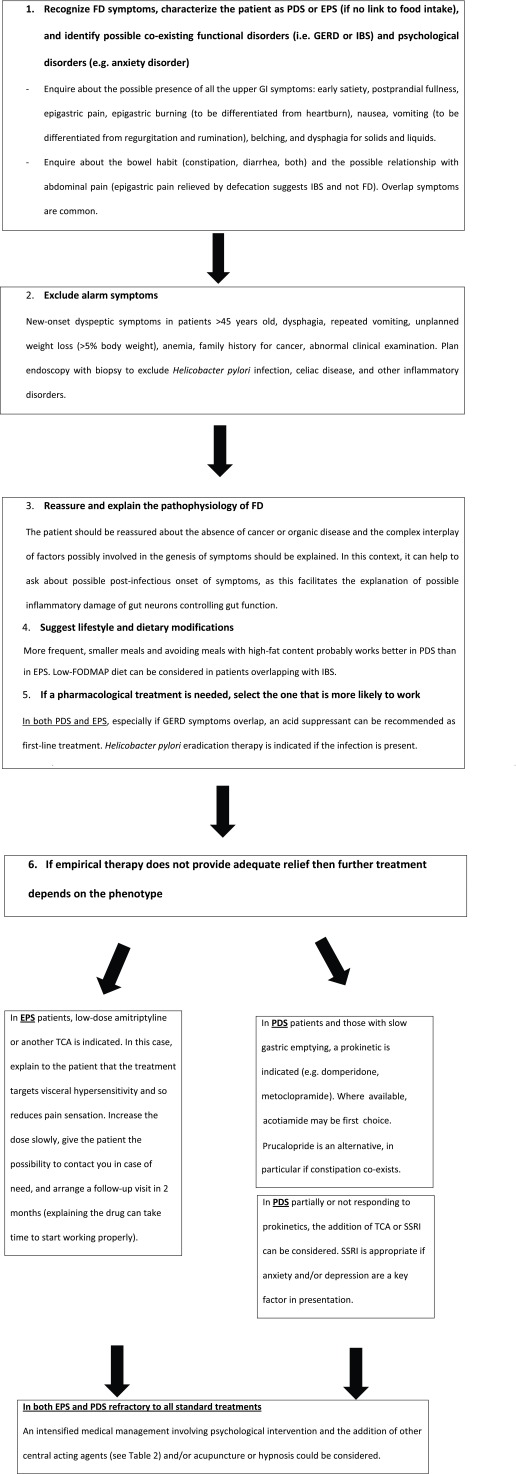
Practical management algorithm in functional dyspepsia. EPS, epigastric pain syndrome; FD, functional dyspepsia; FODMAP, fermentable oligosaccharides, disaccharides, monosaccharides, and polyols; GERD, gastro-oesophageal reflux disease; GI, gastrointestinal; IBS, irritable bowel syndrome; PDS, postprandial distress syndrome; SSRI, selective serotonin reuptake inhibitor; TCA, tricyclic antidepressant.

The authors of the present review work as clinical gastroenterologists with a specialist research interest in functional GI disorders and with experience in different European countries. They consider that the recent literature provides information that can inform and hopefully improve clinical practice. Table 2 and Figure 1, respectively, report the pharmacological options and the practical management algorithm in FD.

**Table 2.** Pharmacological treatment options for functional dyspepsia.

**Acid suppression** Proton pump inhibitors (standard dose should be enough) H2 receptor antagonists (less effective acid suppression than proton pump inhibitors; however, anti-histamine effects may be beneficial in some patients with functional dyspepsia)
**Prokinetic** Metoclopramide (limited evidence and not recommended for long-term treatment because of neurological side effects) Domperidone (limited evidence and not recommended for long-term treatment because of potential cardiac arrhythmias) Prucalopride (evidence based on one study published as abstract) Acotiamide (evidence based on two phase III studies, currently available only in Japan)
**Centrally acting drugs** Tricyclic antidepressants (for example, low-dose amitriptyline 25 mg daily, thought to reduce visceral hypersensitivity and reduce functional pain) Selective serotonin reuptake inhibitors (for example, paroxetine, primarily targeting co-existing anxiety and depression) Neuroleptics (for example, levosulpiride also has prokinetic effects) 5-HT1 agonists (that is, buspirone, tandospirone anxiolytics that may also improve early satiety and postprandial distress) Noradrenergic and specific serotonergic antidepressants (for example, mirtazapine has anxiolytic effects and may also relax the gastric fundus, reduce early satiety, and improve appetite)
**Herbal preparations** Iberogast (no prescription required, may have prokinetic effects on gastric emptying with improvement in reflux and dyspeptic symptoms) Rikkunshito (may have prokinetic effects and improve dyspeptic symptoms)

Whenever we consider the primary, secondary, or tertiary setting, it is of key importance to recognize the typical presentation of patients with FD. The Rome criteria are not primarily intended for use in clinical practice; however, it can be helpful to use symptom-based criteria to establish a “positive” diagnosis of this condition. This aids communication with the patient. At the same time, the criteria can help to differentiate FD from other conditions (for example, GERD alone, IBS alone, or chronic nausea and vomiting disorders). If alarm symptoms are present, then these should be investigated (even if there is a high suspicion of functional disease). If they are absent, then endoscopy is not mandatory at the initial presentation and non-invasive investigation is recommended to test for the presence of *H. pylori* and, if present, to eradicate it, regardless of the presence of different subgroups of patients with FD. Resolution of symptoms after eradication should be reassessed in a few months. In patients with a negative test for *H. pylori* or without resolution of symptoms after eradication, the symptoms of FD should be carefully evaluated to identify those with EPS, PDS, and overlap, which according to the recent publication should be considered PDS ^3^.

EPS should be treated with acid suppressants. If PPI therapy is not effective, then some patients may benefit more from H2RAs ^24^. An alternative (or supplementary) therapy is the addition of an alginate preparation, recently re-evaluated and found to be effective in the treatment of GERD ^26^.

In patients with EPS, if a trial of acid and reflux suppression fails, then tricyclic antidepressants, such as amitriptyline, should be considered. In clinical practice, we start with slow increasing of the dose (normally amitriptyline 25 mg one quarter daily for 1 week, then one half daily for another week, and finally one daily until the next consultation in about 2 months), informing the patient about possible delayed benefit and early minor side effects (that is, dry mouth). We find this approach useful to minimize side effects and improve compliance. Moreover, a follow-up consultation at about 2 months reinforces the patient’s motivation to continue the treatment.

In contrast, in patients with PDS, prokinetic therapy may improve symptoms. However, as summarized above, the market does not offer much choice. Taken on an as-required basis, domperidone or metoclopramide is a reasonable first choice for this patient group; however, long-term treatment is no longer recommended. The authors of the present review have obtained good clinical responses applying prucalopride in some patients with FD, as recently suggested by the literature. Starting with half the licenced dose for constipation (prucalopride 1 mg daily) and building up to 2 mg daily if tolerated is a useful addition, in particular in FD patients with overlapping constipation. In PDS patients who fail to respond, treatment with an antidepressant is the next step; however, it may be reasonable to check gastric emptying prior to starting treatment because the treatment response appears to be poor if delayed gastric emptying is present ^36^. In severe cases, especially those with weight loss, mirtazapine can be a good choice since it also relaxes the stomach and promotes weight gain ^40^. Given the frequent association with anxiety, if this is considered a likely driver of symptoms, then treatment with SSRIs is reasonable, even though no data support this approach in FD patients per se. Certainly, anxiety and depression have been shown to impact on gastric motor and sensory function ^41^.

In patients who do not respond to empirical treatment, further functional evaluations are normally advocated to select appropriate management ^1^. However, many of these investigations are available in highly specialized referral centres or used primarily for research purposes or both ^42^. The electronic barostat is considered the gold standard to assess gastric accommodation and visceral sensitivity, but the technique is invasive and is mainly used for research, and recent studies have suggested that the barostat bag could influence the gastric motor response ^42^. New, less invasive techniques are in evaluation to replace the barostat. The nutrient drink test has been proposed as an alternative. In this test, a defined nutrient meal is ingested over a period of time until dyspeptic symptoms develop or the patient reports feeling full ^42^. Response to treatment can be measured by repeating the test ^42^. More recently, intragastric pressure measurement during a nutrient meal change was proposed as a minimally invasive test of nutrient tolerance and accommodation in FD ^42^. However, the test needs further validation ^42^. Gastric scintigraphy is the reference test of gastric emptying and is clinically more widely accessible than tests of accommodation ^43^. Methodology has been extensively validated; however, unfortunately, standard test meals are not widely applied ^43^. In most centres, only the gastric emptying of solids is evaluated ^43^. If gastric emptying is delayed, then the diagnosis of “gastroparesis” is made; however, unless the delay is severe (for example, three times the upper limit of normal), there is only a weak association between this finding and patient symptoms or disease severity ^44^. Moreover, it is not clear that the finding of gastroparesis predicts positive outcome for prokinetic medication ^44^. Conversely, the diagnosis of gastric dumping has implications for dietary and pharmacological therapy ^44^. It is interesting to note that patients with rapid gastric emptying (that is, dumping) can present with symptoms identical to those of gastroparesis and this may be the strongest reason to do the test ^44^.

However, the reality is that after performing tests, at this time, not so many specific pharmacological therapies are available to correct abnormal physiology ^1^. Nevertheless, there is evidence that a clear explanation for symptoms improves patient acceptance of disease and reduces out-patient attendance ^45^. Otherwise, an intensified medical management involving psychological intervention could be applied, as this has been reported to give superior long-term outcomes ^46^.

**Figure 2.**
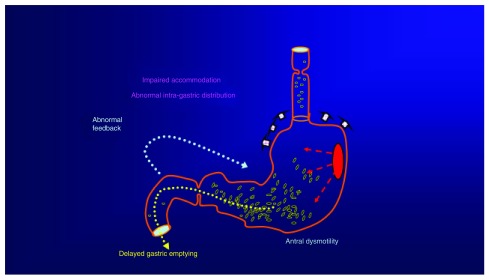
Abnormal gastric motility and function could be implicated in the pathophysiology of symptoms in functional dyspepsia, especially in patients with postprandial distress syndrome. Heightened visceral sensitivity has an important role in epigastric pain syndrome but is also present in many individuals with postprandial distress. In clinical practice, with the exception of detection of abnormal gastric emptying by scintigraphy, it is currently not possible to identify the specific causes of disease.

At present, the information obtained from gastric emptying studies is limited. For example, gastric scintigraphy studies often provide only a single measurement of gastric emptying of a solid meal. As already suggested ^43^, it is unlikely that this captures the complexity of GI function ( Figure 2). In particular, a single summary outcome measurement, normalized after ingestion of a meal, misses the “early gastric emptying” that occurs even during meal ingestion ^47^. Moreover, the current way of performing the gastric emptying test does not incorporate the report of sensations experienced during meal ingestion ^47^. A new development in this field is the combination of a nutrient drink test with gastric scintigraphy to obtain a comprehensive assessment of gastric motor and sensory function ^47^. Assessment of the scintigraphic images after ingestion of the relatively large (400 mL) “Nottingham Test Meal” provides an evaluation of gastric accommodation and sensitivity in addition to gastric emptying ^47^. The test uses standard technology, and normal values of this test have been published ^48^. Ongoing studies will disclose whether this test can predict the response to pharmacological treatments in clinical practice.

## Conclusions

The literature of these last five years concerning FD has provided some relevant information for clinical practice. It has indeed confirmed the importance of correctly recognizing the different symptoms affecting the patient in order to identify PDS or EPS. This classification indeed seems to correspond to different pathophysiological mechanisms and treatments. However, it also reveals that we urgently need new tests to better study the GI function of these patients in order to develop effective treatments and better understand the pathophysiological mechanisms to target.

## Abbreviations

CHS, cannabinoid hyperemesis syndrome; CNVS, chronic nausea and vomiting syndrome; CVS, cyclic vomiting syndrome; EPS, epigastric pain syndrome; ERD, erosive reflux disease; FD, functional dyspepsia; FH, functional heartburn; GERD, gastro-oesophageal reflux disease; GI, gastrointestinal; IBS, irritable bowel syndrome; NERD, non-erosive reflux disease; PDS, postprandial distress syndrome; PPI, proton pump inhibitor; SNRI, serotonin-norepinephrine reuptake inhibitor; SSRI, selective serotonin receptor inhibitor.
